# Male sex is not an independent risk factor for recurrence of differentiated thyroid cancer: a propensity score-matching study

**DOI:** 10.1038/s41598-021-94461-5

**Published:** 2021-07-21

**Authors:** Joonseon Park, Kwangsoon Kim, Dong-Jun Lim, Ja Seong Bae, Jeong Soo Kim

**Affiliations:** 1 Department of Surgery, College of Medicine, The Catholic University of Korea, 06591 Seoul, Republic of Korea; 2 Division of Endocrinology and Metabolism, Department of Internal Medicine, College of Medicine, The Catholic University of Korea, 06591 Seoul, Republic of Korea

**Keywords:** Cancer, Endocrinology

## Abstract

Male patients have a significantly higher prevalence of advanced-stage thyroid cancer. However, sex differences in the risk of differentiated thyroid carcinoma (DTC) recurrence have not been fully elucidated. Therefore, the present study aimed to investigate male sex as a prognostic factor for DTC. We assessed 5566 patients with DTC who underwent thyroid surgery between January 2009 and December 2015 at Seoul St. Mary’s Hospital (Seoul, Korea). Clinicopathological characteristics and long-term oncologic outcomes between female and male patients with DTC were compared using propensity score matching to reduce selection bias. The mean follow-up duration was 99.9 ± 18.7 months. The recurrence rate was significantly higher in male patients than female patients before matching (3.3% vs. 2.2%, *p* = 0.030), and there was no significant difference in recurrence rates between the matched groups after matching (3.0% vs. 2.5%, *p* = 0.591). Based on Kaplan–Meier analysis, the two groups did not significantly differ in disease-free survival after matching. Multivariate analysis revealed that male sex was not an independent prognostic factor of DTC recurrence. Male sex did not have a significant effect on DTC recurrence. Further studies with larger cohorts are required to validate the findings of this study.

## Introduction

Thyroid cancer is one of the most common endocrine malignancies. The incidence of thyroid cancer has substantially increased worldwide in the last several decades^1–3^. Differentiated thyroid carcinoma (DTC), including papillary thyroid carcinoma (PTC), follicular thyroid carcinoma (FTC), and Hürthle cell carcinoma, is the most common malignancy of the thyroid gland^4^. In terms of overall incidence, DTC accounts for 1%–1.5% of all cases of cancer, and its incidence is three times higher in female patients than male patients^2,5,6^.

Generally, DTC has an excellent prognosis because of its indolent features. According to a Surveillance, Epidemiology, and End Results report, patients with DTC have an overall survival rate of 90%–95%^7^. However, some clinicopathological risk factors, including age, tumor size, lymph node (LN) metastasis, and extrathyroidal extension (ETE), are associated with the aggressive nature and high recurrence of DTC^8^. The American Thyroid Association (ATA) management guidelines have proposed a clinicopathological risk stratification system that can be used for classifying patients as those who are at low, intermediate, or high risk. Using this system, several factors, such as ETE, LN metastasis, aggressive histology, vascular invasion, and multifocality were found to be associated with increased risk of recurrence^9^. However, sex is not considered a risk factor for recurrence.

DTC is more prevalent in women than men but the cause is not fully elucidated^10^. Li et al. reported that male patients had a 0.78-fold risk of developing malignancy and larger tumor size than female patients^11^. Moreover, male patients had a significantly higher prevalence of advanced-stage thyroid cancer, LN metastasis, and ETE^12^. Some studies have reported that sex, age, tumor size, aggressive histology, ETE, and LN metastasis were significant prognostic factors for thyroid cancer^13–17^. The impact of sex on the risk of DTC recurrence is controversial, and results pertaining to this concept vary in the literature^14,16,18–21^. Toniato et al. and several other studies have concluded that sex does not significantly contribute to recurrence and survival^16,20^. By contrast, Zahedi et al. studied patients with DTC who were > 18 years old and showed that the recurrence rate was higher in males than in females, a finding supported by other studies^14,18,19^. In another novel study, Choi H. et al. summarized secular trends by analyzing results to date and found that the poor outcome of PTC associated with men decreased over time, whereas aggressive pathological features remained the same or increased over time^21^.

Therefore, the current study aimed to compare the clinicopathological characteristics and long-term oncologic outcomes between females and males with DTC using propensity score matching to reduce selection bias.

## Results

### Comparison of clinicopathological characteristics between female and male patients before and after propensity score matching

Table 1 shows the baseline clinicopathological characteristics of female and male patients before and after propensity score matching. In terms of age, males were significantly younger than females (45.8 ± 11.8 vs. 46.8 ± 12.2 years, *p* = 0.017). The surgical extent was significantly more extensive in males than females (*p* = 0.001). Tumor size was significantly larger in males than females (1.1 ± 0.8 vs. 0.9 ± 0.7 cm, *p* < 0.001). The incidence of lymphatic and vascular invasions was significantly higher in males than females (34.3% vs. 24.6%, *p* < 0.001 and 4.0% vs. 1.9%, *p* < 0.001, respectively). Female patients had a significantly higher prevalence of ETE that male patients (5.7% vs. 3.9%. *p* = 0.022). Moreover, the proportion of harvested and positive LNs was higher in males than females (12.6 ± 16.7 vs. 10.7 ± 12.1, *p* < 0.001 and 3.1 ± 4.9 vs. 1.7 ± 3.4, *p* < 0.001, respectively). Males had significantly more advanced N and TNM stage than females (*p* < 0.001 and *p* = 0.021, respectively). A significantly higher recurrence rate was observed in males than females before matching (3.3% vs. 2.2%, *p* = 0.030).

**Table 1:** Comparison of clinicopathological characteristics between females and males before and after propensity score matching.

	Before matching			After matching		
	Female (n = 4420)	Male (n = 1146)	p-value	Female (n = 1040)	Male (n = 1040)	p-value
**Age (years)**	46.8 ± 12.2 (range, 12–88)	45.8 ± 11.8 (range, 11–81)	0.017	46.5 ± 12.4 (range, 14–79)	46.2 ± 11.8 (range, 11–81)	0.535
**Extent of operation**			0.001			0.376
Less than TT	3313 (75.0%)	805 (70.2%)		764 (73.5%)	745 (71.6%)	
TT and/or mRND	1107 (25.0%)	341 (29.8%)		276 (26.5%)	295 (28.4%)	
**Type of carcinoma**			0.823			0.986
PTC	4333 (98.0%)	1124 (98.1%)		1018 (97.9%)	1019 (98.0%)	
FTC	75 (1.7%)	18 (1.6%)		18 (1.7%)	17 (1.6%)	
HTC	12 (0.3%)	4 (0.3%)		4 (0.4%)	4 (0.4%)	
**Tumor size (cm)**	0.9 ± 0.7 (range, 0.2–9.0)	1.1 ± 0.8 (range, 0.2–5.5)	< 0.001	1.1 ± 0.8 (range, 0.2–6.5)	1.1 ± 0.8 (range, 0.2–5.5)	0.933
**ETE**	250 (5.7%)	45 (3.9%)	0.022	39 (3.8%)	40 (3.8%)	0.909
**Multifocality**	1708 (38.6%)	446 (38.9%)	0.865	398 (38.3%)	390 (37.5%)	0.752
**Bilaterality**	414 (9.4%)	101 (8.8%)	0.607	101 (9.7%)	82 (7.9%)	0.163
**Lymphatic invasion**	1086 (24.6%)	393 (34.3%)	< 0.001	326 (31.3%)	325 (31.3%)	0.962
**Vascular invasion**	83 (1.9%)	46 (4.0%)	< 0.001	35 (3.4%)	35 (3.4%)	1.000
**Perineural invasion**	81 (1.8%)	24 (2.1%)	0.544	21 (2.0%)	20 (1.9%)	0.875
**BRAF**^**V600E**^** positive**	2871/3650 (78.7%)	748/929 (80.5%)	0.223	659/866 (76.1%)	675/846 (79.8%)	0.071
**Harvested LNs**	10.7 ± 12.1	12.6 ± 16.7	< 0.001	10.8 ± 12.3	11.7 ± 15.2	0.109
**Positive LNs**	1.7 ± 3.4	3.1 ± 4.9	< 0.001	2.4 ± 3.8	2.6 ± 3.9	0.355
**T stage**			0.710			0.779
T1	3911 (88.5%)	991 (86.5%)		924 (88.8%)	909 (87.4%)	
T2	218 (4.9%)	91 (7.9%)		64 (6.2%)	74 (7.1%)	
T3a	41 (0.5%)	19 (1.7%)		13 (1.3%)	17 (1.6%)	
T3b	238 (5.4%)	44 (3.8%)		37 (3.6%)	39 (3.8%)	
T4a	12 (0.3%)	1 (0.1%)		2 (0.2%)	1 (0.1%)	
**N stage**			< 0.001			0.502
N0	2351 (53.2%)	471 (41.1%)		456 (43.8%)	455 (43.8%)	
N1a	1880 (42.5%)	597 (52.1%)		533 (51.3%)	522 (50.2%)	
N1b	189 (4.3%)	78 (6.8%)		51 (4.9%)	63 (6.1%)	
**M stage**			0.471			
M1	2 (0.0%)	0 (0.0%)		0 (0%)	0 (0%)	
**TNM stage**			0.021			0.530
Stage I	3903 (88.3%)	983 (85.8%)		887 (85.3%)	898 (86.3%)	
Stage II	516 (11.7%)	163 (14.2%)		153 (14.7%)	142 (13.7%)	
Stage III	1 (0.0%)	0 (0%)		0 (0%)	0 (0%)	0.530
**Recurrence**	96 (2.2%)	38 (3.3%)	0.030	26 (2.5%)	31 (3.0%)	0.591

Data are expressed as patient’s number (%), or mean ± SD.

A statistically significant difference was defined as p < 0.05.

*TT* total thyroidectomy, *mRND* modified radical neck dissection, *PTC* papillary thyroid carcinoma, *FTC* follicular thyroid carcinoma, *HTC* Hürthle cell thyroid carcinoma, *ETE* extrathyroidal extension, *LN* lymph node, *T* tumor, *N* node, *M* metastasis.

Propensity score matching yielded 1040 matched pairs of patients. There were no significant differences in clinicopathological characteristics, including the recurrence rate, between the matched groups (Table 1).

### Univariate and multivariate analyses of the risk factors for recurrence before and after propensity score matching

Table 2 presents the univariate and multivariate Cox regression analysis results for the risk factors of recurrence before propensity score matching. Age ≤ 45 years (hazard ratio (HR) 1.841; 95% confidence interval (CI) 1.278–2.652; *p* = 0.001), ETE (HR 2.196; 95% CI 1.305–3.695; *p* = 0.003), vascular invasion (HR 2.504; 95% CI 1.286–4.876; *p* = 0.007), and number of positive LNs were considered to be significant predictors of recurrence (HR 1.062; 95% CI 1.041–1.083; *p* < 0.001). Among the various risk factors, N1a stage was the most significant predictor of recurrence (HR 3.159; 95% CI 2.015–4.955; *p* < 0.001).

**Table 2:** Univariate and multivariate analyses of risk factors for recurrence before propensity score matching.

	Univariate		Multivariate	
	HR (95% CI)	p-value	HR (95% CI)	p-value
**Gender**				
Female	Ref.			
Male	1.552 (1.066–2.259)	0.022		
**Age (years)**				
> 45	Ref.		Ref.	
≤ 45	2.192 (1.537–3.125)	< 0.001	1.841 (1.278–2.652)	0.001
**Extent of operation**				
Less than TT	Ref.			
TT and/or mRND	1.821 (1.286–2.577)	0.001		
**Tumor size**				
< 1 cm	Ref.			
≥ 1 cm	2..136 (1.522–2.998)	< 0.001		
**ETE**	3.409 (2.140–5.432)	< 0.001	2.196 (1.305–3.695)	0.003
**Multifocality**	1.563 (1.114–2.193)	0.010		
**Bilaterality**	1.744 (1.084–2.805)	0.022		
**Lymphatic invasion**	3.202 (2.281–4.496)	< 0.001		
**Vacular invasion**	3.471 (1.822–6.611)	< 0.001	2.504 (1.286–4.876)	0.007
**Perineural invasion**	3.335 (1.632–6.815)	0;001		
**Harvested LNs**	1.021 (1.014–1.028)	< 0.001		
**Positive LNs**	1.076 (1.063–1.088)	< 0.001	1.062 (1.041–1.083)	< 0.001
**T stage**				
T1	Ref.			
T2	2.488 (1.445–4.285)	0.001		
T3b	3.649 (2.256–5.903)	< 0.001		
T4a	3.928 (0.548–28.173)	0.173		
**N stage**				
N0	Ref.		Ref.	
N1a	4.350 (2.797–6.765)	< 0.001	3.159 (2.015–4.955)	< 0.001
N1b	7.002 (3.738–13.116)	< 0.001	1.488 (0.630–3.512)	0.364

Data are expressed as hazard ratio (HR) and 95% confidence interval (CI).

A p value < 0.05 was considered statistically significant.

*TT* total thyroidectomy, *mRND* modified radical neck dissection, *ETE* extrathyroidal extension, *LN* lymph node, *T* tumor, *N* node.

**Figure 1:**
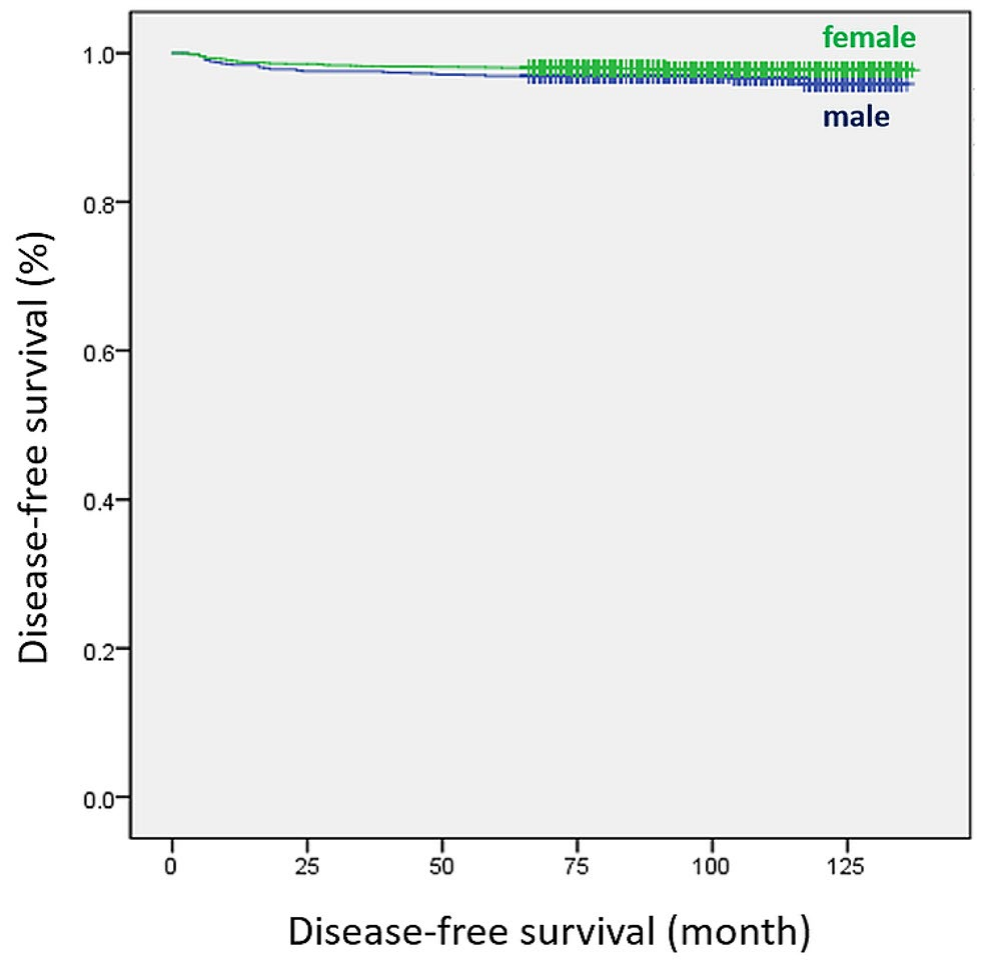
Disease-free survival curves of female and male groups before propensity score matching (log-rank *p* = 0.021).

**Figure 2:**
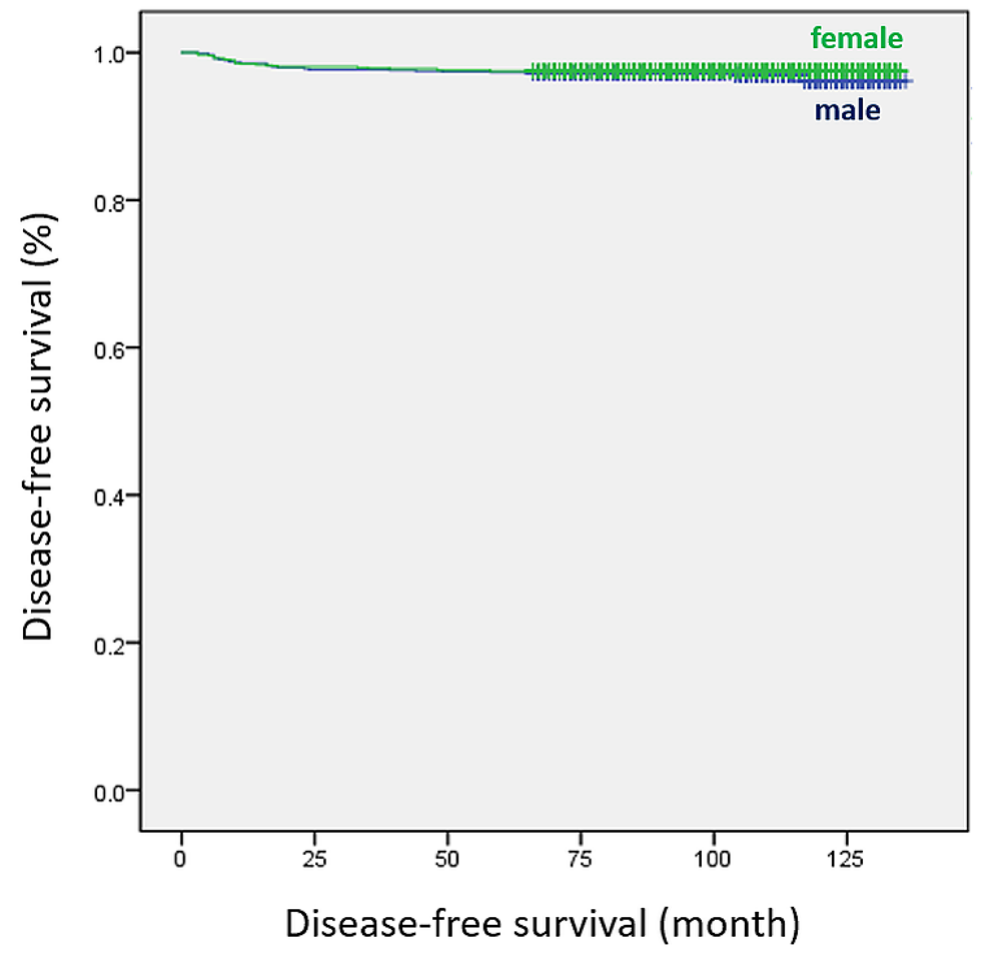
Disease-free survival curves of female and male groups after propensity score matching (log-rank *p* = 0.492).

After propensity score matching, the number of positive LNs (HR 1.193; 95% CI 1.129–1.260; *p* < 0.001) and N1a stage (HR 2.401; 95% CI 1.010–5.708; *p* = 0.047) were found to be significant predictors of recurrence (Table 3). In the Kaplan–Meier analysis, disease-free survival (DFS) was significantly differed between the two groups before matching (*p* = 0.021, Fig. 1). However, there was no significant difference in DFS between the two groups after matching (*p* = 0.492, Fig. 2).

**Table 3:** Univariate and multivariate analyses of risk factors for recurrence after propensity score matching.

	Univariate		Multivariate	
	HR (95% CI)	p-value	HR (95% CI)	p-value
**Tumor size**				
< 1 cm	Ref.			
≥ 1 cm	2.155 (1.281–3.624)	0.004		
**ETE**	4.330 (2.050–9.144)	< 0.001		
**Bilaterality**	2.259 (1.141–4.471)	0.019		
**Lymphatic invasion**	4.185 (2.429–7.210)	< 0.001		
**Perineural invasion**	3.787 (1.370–10.464)	0.010		
**Harvested LNs**	1.022 (1.012–1.032)	< 0.001		
**Positive LNs**	1.152 (1.115–1.190)	< 0.001	1.193 (1.129–1.260)	< 0.001
**T stage**				
T1	Ref.			
T2	3.079 (1.494–6.346)	0.002		
T3b	5.077 (2.376–10.847)	< 0.001		
**N stage**				
N0	Ref.		Ref.	
N1a	5.432 (2.444–12.077)	< 0.001	2.401 (1.010–5.708)	0.047
N1b	8.255 (2.895–23.542)	< 0.001	0.545 (0.121–2.464)	0.431

Data are expressed as hazard ratio (HR) and 95% confidence interval (CI).

A p value < 0.05 was considered statistically significant.

*ETE* extrathyroidal extension, *LN* lymph node, *T* tumor, *N* node.

### Subgroup analysis between female and male patients aged between 20 and 45 years before and after propensity score matching

The baseline clinicopathological characteristics of female and male patients aged between 20 and 45 years are summarized in Table 4. Before propensity score matching, age was considered to be a significant factor. That is, males were significantly older than females (36.8 ± 5.2 vs. 35.8 ± 6.2 years, *p* = 0.001). Moreover, males underwent significantly more extensive surgeries than females (*p* = 0.001). The incidence of lymphatic invasion was significantly higher in males than in females (36.8% vs. 30.3%, *p* = 0.003). Male patients had a significantly higher proportion of harvested and positive LNs than female patients (13.9 ± 16.9 vs. 11.4 ± 13.0, *p* < 0.001 and 3.6 ± 5.3 vs. 2.2 ± 3.8, *p* < 0.001, respectively). N stage was significantly more advanced in males than females (*p* < 0.001). However, there were no significant differences in recurrence rates between the two groups (*p* = 0.086).

**Table 4:** Subgroup comparison between female and male participants aged 20–45 years before and after propensity score matching.

	Before matching			After matching		
	Female (n = 1948)	Male (n = 587)	p-value	Female (n = 515)	Male (n = 515)	p-value
**Age (years)**	35.8 ± 6.2 (range, 20–45)	36.8 ± 5.2 (range, 20–45)	0.001	36.8 ± 6.0 (range, 20–45)	36.7 ± 5.2 (range, 20–45)	0.722
**Extent of operation**			0.001			0.946
Less than TT	1470 (75.5%)	402 (68.5%)		356 (69.1%)	355 (68.9%)	
TT and/or mRND	478 (24.5%)	185 (31.5%)		159 (30.9%)	160 (31.1%)	
**Type of carcinoma**			0.283			0.155
PTC	1915 (98.3%)	576 (98.1%)		510 (99.0%)	504 (97.9%)	
FTC	30 (1.5%)	8 (1.4%)		5 (1.0%)	8 (1.6%)	
HTC	3 (0.2%)	3 (0.5%)		0 (0%)	3 (0.6%)	
**Tumor size (cm)**	1.0 ± 0.7 (range, 0.2–9.0)	1.0 ± 0.7 (range, 0.2–5.5)	0.072	1.0 ± 0.8 (range, 0.2–9.0)	1.0 ± 0.7 (range, 0.2–5.5)	0.589
**ETE**	71 (3.6%)	19 (3.2%)	0.704	20 (3.9%)	18 (3.5%)	0.869
**Multifocality**	653 (33.5%)	206 (35.1%)	0.486	174 (33.8%)	172 (33.4%)	0.947
**Bilaterality**	156 (8.0%)	47 (8.0%)	0.999	50 (9.7%)	37 (7.2%)	0.178
**Lymphatic invasion**	590 (30.3%)	216 (36.8%)	0.003	186 (36.1%)	180 (35.0%)	0.745
**Vascular invasion**	40 (2.1%)	18 (3.1%)	0.157	15 (2.9%)	15 (2.9%)	1.000
**Perineural invasion**	26 (1.3%)	11 (1.9%)	0.330	7 (1.4%)	9 (1.7%)	0.802
**BRAF**^**V600E**^** positive**	1250/1608 (77.7%)	388/476 (81.5%)		322/416 (77.4%)	337/417 (80.8%)	0.234
**Harvested LNs**	11.4 ± 13.0	13.9 ± 16.9	< 0.001	13.0 ± 15.2	13.1 ± 15.4	0.798
**Positive LNs**	2.2 ± 3.8	3.6 ± 5.3	< 0.001	3.2 ± 5.1	3.1 ± 4.5	0.816
**T stage**			0.219			0.748
T1	1745 (89.6%)	519 (88.4%)		460 (89.3%)	455 (88.3%)	
T2	116 (6.0%)	47 (8.0%)		32 (6.2%)	40 (7.8%)	
T3a	16 (0.8%)	2 (0.3%)		3 (0.6%)	2 (0.4%)	
T3b	67 (3.4%)	19 (3.2%)		20 (3.9%)	18 (3.5%)	
T4a	4 (0.2%)	0 (0%)		0 (0%)	0 (0%)	
**N stage**			< 0.001			0.847
N0	921 (47.3%)	210 (35.8%)		194 (37.7%)	197 (38.3%)	
N1a	917 (47.1%)	327 (55.7%)		277 (53.8%)	279 (54.2%)	
N1b	110 (5.6%)	50 (8.5%)		44 (8.5%)	39 (7.6%)	
**TNM stage**						1.000
Stage I	1946 (99.9%)	587 (100%)	0.590	514 (99.8%)	515 (100%)	
Stage II	2 (0.1%)	0 (0%)		1 (0.2%)	0 (0%)	
**Recurrence**	63 (3.2%)	21 (3.6%)	0.086	23 (4.5%)	20 (3.9%)	0.756

Data are expressed as patient’s number (%), or mean ± SD.

A statistically significant difference was defined as p < 0.05.

*TT* total thyroidectomy, *mRND* modified radical neck dissection, *PTC* papillary thyroid carcinoma, *FTC* follicular thyroid carcinoma, *HTC* Hürthle cell thyroid carcinoma, *ETE* extrathyroidal extension, *LN* lymph node, *T* tumor, *N* node, *M* metastasis.

Propensity score matching yielded 515 matched pairs of patients. There were no significant differences in clinicopathological characteristics between the matched groups (Table 4).

Table 5 depicts the risk factors for recurrence among patients aged between 20 and 45 years after propensity score matching. Lymphatic invasion (HR 2.214; 95% CI 1.118–4.381; *p* = 0.023) and the proportion of positive LNs (HR 1.071; 95% CI 1.028– 1.115; *p* < 0.001) were considered to be significant predictors of recurrence.

**Table 5:** Univariate and multivariate analyses of risk factors for recurrence in patients aged 20–45 years after propensity score matching.

	Univariate		Multivariate	
	HR (95% CI)	p-value	HR (95% CI)	p-value
**Extent of operation**				
Less than TT	Ref.			
TT and/or mRND	2.380 (1.309–4.329)	0.004		
**ETE**	2.916 (1.042–8.163)	0.042		
**Bilaterality**	2.259 (1.141–4.471)	0.019		
**Lymphatic invasion**	3.217 (1.732–5.972)	< 0.001	2.214 (1.118–4.381)	0.023
**Vascular invasion**	4.446 (1.748–11.305)	0.002		
**Harvested LNs**	1.015 (1.001–1.029)	0.041		
**Positive LNs**	1.094 (1.056–1.133)	< 0.001	1.071 (1.028–1.115)	< 0.001
**N stage**				
N0	Ref.			
N1a	3.148 (1.383–7.169)	0.006		
N1b	4.255 (1.429–12.671)	0.009		

Data are expressed as hazard ratio (HR) and 95% confidence interval (CI).

A p value < 0.05 was considered statistically significant.

*TT* total thyroidectomy, *mRND* modified radical neck dissection, *ETE* extrathyroidal extension, *LN* lymph node, *N* node.

### Subgroup analysis between female and male patients aged over 45 years before and after propensity score matching

Table 6 presents the baseline clinicopathological characteristics of females and males aged over 45 years before and after propensity score matching. The tumor size was significantly larger in males than in females (1.1 ± 0.9 vs. 1.0 ± 0.7 cm, *p* < 0.001). Males had a significantly higher prevalence of ETE than females (4.5% vs 7.5%, *p* < 0.024). The incidence of lymphatic and vascular invasions was significantly higher in males than in females (31.2% vs. 19.7%, *p* < 0.001 and 4.5% vs. 1.7%, *p* < 0.001, respectively). Males had a significantly higher proportion of positive LNs than females (2.4 ± 4.2 vs. 1.3 ± 2.6, *p* < 0.001). Further, male patients had a significantly higher T, N, and TNM stage than female patients (*p* < 0.001, *p* < 0.001 and *p* < 0.001, respectively). The recurrence rate was significantly higher in males than in females (2.9% vs 1.3%, *p* = 0.012). After matching, the recurrence rate did not significantly differ between the two groups (*p* = 0.301).

**Table 6:** Subgroup comparison between females and males aged > 45 years before and after propensity score matching.

	Before matching			After matching		
	Female (n = 2451)	Male (n = 552)	p-value	Female (n = 515)	Male (n = 515)	p-value
**Age (years)**	55.8 ± 7.3 (range, 46—88)	55.9 ± 7.8 (range, 46—81)	0.796	56.3 ± 7.5 (range, 46—83)	55.8 ± 7.8 (range, 46—81)	0.360
**Extent of operation**			0.259			0.003
Less than TT	1829 (74.6%)	399 (72.3%)		401 (82.9%)	362 (74.8%)	
TT and/or mRND	622 (25.4%)	153 (27.7%)		83 (17.1%)	122 (25.2%)	
**Type of carcinoma**			0.774			0.035
PTC	2399 (97.9%)	542 (98.2%)		461 (95.2%)	475 (98.1%)	
FTC	43 (1.8%)	9 (1.6%)		18 (3.7%)	8 (1.7%)	
HTC	9 (0.4%)	1 (0.2%)		5 (1.0%)	1 (0.2%)	
**Tumor size (cm)**	1.0 ± 0.7 (range, 0.2 – 6.0)	1.1 ± 0.9 (range, 0.2 – 5.5)	< 0.001	1.1 ± 0.9 (range, 0.2 – 5.0)	1.1 ± 0.9 (range, 0.2 – 5.5)	0.906
**ETE**	177 (7.2%)	25 4.5%)	0.024	19 (3.9%)	22 (4.5%)	0.750
**Multifocality**	1049 (42.8%)	239 (42.3%)	0.849	215 (44.4%)	205 (42.4%)	0.559
**Bilaterality**	256 (10.4%)	53 (9.6%)	0.588	42 (8.7%)	45 (9.3%)	0.822
**Lymphatic invasion**	483 (19.7%)	172 (31.2%)	< 0.001	120 (24.8%)	131 (27.1%)	0.463
**Vascular invasion**	41 (1.7%)	25 (4.5%)	< 0.001	22 (4.5%)	17 (3.5%)	0.514
**Perineural invasion**	54 (2.2%)	13 (2.4%)	0.873	11 (2.3%)	11 (2.3%)	1.000
**BRAF**^**V600E**^** positive**	1611/2026 (79.5%)	354/447 (79.2%)	0.897	325/402 (80.8%)	313/394 (79.4%)	0.657
**Harvested LNs**	10.1 ± 10.8	10.9 ± 14.6	0.151	9.9 ± 11.6	10.9 ± 15.1	0.230
**Positive LNs**	1.3 ± 2.6	2.4 ± 4.2	< 0.001	2.0 ± 3.4	2.3 ± 4.2	0.313
**T stage**			< 0.001			0.879
T1	2153 (87.8%)	470 (85.1%)		423 (87.4%)	418 (88.3%)	
T2	97 (4.0%)	41 (7.4%)		31 (6.4%)	33 (7.8%)	
T3a	24 (1.0%)	16 (2.9%)		11 (2.3%)	11 (0.4%)	
T3b	169 (6.9%)	24 (4.3%)		19 (3.9%)	21 (3.5%)	
T4a	8 (0.3%)	1 (0.2%)		0 (0%)	1 (0.2%)	
**N stage**			< 0.001			0.909
N0	1425 (58.1%)	260 (47.1%)		250 (51.7%)	244 (50.4%)	
N1a	951 (38.8%)	265 (48.0%)		210 (43.4%)	214 (44.2%)	
N1b	75 (3.1%)	27 (4.9%)		24 (5.0%)	26 (5.4%)	
**TNM stage**			< 0.001			0.884
Stage I	1936 (79.0%)	389 (70.5%)		356 (73.6%)	359 (74.2%)	
Stage II	514 (21.0%)	163 (29.5%)		128 (26.4%)	125 (25.8%)	
Stage III	1 (0.0%)	0 (0%)		0 (0%)	0 (0%)	
**Recurrence**	31 (1.3%)	16 (2.9%)	0.012	9 (1.9%)	15 (3.1%)	0.301

Data are expressed as patient’s number(%), or mean ± SD.

A statistically significant difference was defined as p < 0.05.

*TT* total thyroidectomy, *mRND* modified radical neck dissection, *PTC* papillary thyroid carcinoma, *FTC* follicular thyroid carcinoma, *HTC* Hürthle cell thyroid carcinoma, *ETE* extrathyroidal extension, *LN* lymph node, *T* tumor, *N* node, *M* metastasis.

The risk factors for recurrence in patients aged > 45 years after propensity score matching are shown in Table 7. Multifocality (HR 3.438; 95% CI 1.143–10.342; *p* = 0.028) and the proportion of positive LNs (HR 1.259; 95% CI 1.117– 1.429; *p* < 0.001) were found to be significant predictors of recurrence.

**Table 7:** Univariate and multivariate analyses of risk factors for recurrence in patients aged > 45 years after propensity score matching.

	Univariate		Multivariate	
	HR (95% CI)	p-value	HR (95% CI)	p-value
**Tumor size**				
< 1 cm	Ref.			
≥ 1 cm	3.267 (1.430–7.466)	0.005		
**ETE**	7.779 (3.088–19.600)	< 0.001		
**Multifocality**	2.642 (1.131–6.173)	0.025	3.438 (1.143–10.342)	0.028
**Lymphatic invasion**	4.068 (1.807–9.158)	0.001		
**Vascular invasion**	4.810 (1.644–14.072)	0.004		
**Perineural invasion**	8.741 (2.987–25.576)	< 0.001		
**Harvested LNs**	1.027 (1.014–1.040)	< 0.001		
**Positive LNs**	1.112 (1.079–1.147)	< 0.001	1.259 (1.117–1.419)	< 0.001
**T stage**				
T1	Ref.			
T2	3.873 (1.275–11.766)	0.017		
T3b	9.329 (3.585–24.279)	< 0.001		
**N stage**				
N0	Ref.			
N1a	3.545 (1.288–9.754)	0.014		
N1b	8.059 (2.164–30.013)	0.002		

Data are expressed as hazard ratio (HR) and 95% confidence interval (CI).

A p value < 0.05 was considered statistically significant.

*ETE* extrathyroidal extension, *LN* lymph node, *N* node.

## Discussion

DTC is the most common endocrine malignancy, and it usually has a favorable prognosis, with a 10-year disease-specific survival rate of > 90%^2,7,22–24^. However, some patients can develop local recurrence or distant metastasis to the lung and/or bone, resulting in death. This finding indicates that DTC can progress via different clinical pathways. Several clinicopathological factors, including age, tumor size, ETE, multifocality, LN metastasis, and histologic type, were found to be significant prognostic factors for DTC^25–28^. The ATA management guidelines have proposed a risk stratification system based on significant prognostic factors^9^. The American Joint Committee on Cancer/Union for International Cancer Control TNM staging system has been widely used for predicting the prognosis of DTC^29^. However, sex has not been considered a prognostic factor in the commonly used staging systems.

Male sex was associated with a lower DFS and disease-specific survival based on previous studies^18,30–33^. However, some reports showed that the overall survival was not affected by sex^34,35^. A prognostic factor correlated with sex is not a consistent finding, and the effect of sex on DFS has not yet been validated. Recently, Zahedi et al. reported that the risk of DTC recurrence is higher in men than in women^18^. Thus, this study aimed to evaluate the effect of sex on long-term prognosis of DTC.

This study compared the clinicopathological characteristics and long-term oncologic outcomes of male and female patients with DTC. Results showed that compared with females, males had significantly larger tumor sizes, higher prevalence of ETE and lymphatic and vascular invasions and greater proportions of positive LNs and TNM stage. This result is consistent with that of previous studies showing that men were more likely to present with more aggressive disease characteristics^11,12,36,37^. The recurrence rate was significantly higher in males than in females (3.3% vs. 2.2%, *p* = 0.030). However, this result may not be accurate with respect to disease severity.

In this study, the impact of sex was assessed in patients with DTC. However, a significant difference was observed between males and females in terms of several clinicopathological characteristics. That is, males were significantly younger than females. Furthermore, male sex was significantly associated with more aggressive tumor characteristics, including larger tumor size, higher incidence of ETE and lymphatic and vascular invasion, and greater TNM stage and proportion of positive LNs. Therefore, the findings of this study were affected by several confounding factors, including selection bias, between the two groups. To minimize the effects of these factors, propensity score matching analysis was performed to adjust for several clinicopathological characteristics. After matching, the recurrence rates between female and male patient did not significantly differ (2.5% vs. 3.0%, *p* = 0.591).

We performed multivariate Cox regression and Kaplan–Meier analyses to identify whether sex was independently associated with DFS before and after propensity score matching. Before matching, male sex was considered to be a significant predictor of recurrence (HR 1.552; *p* = 0.022) based on the univariate analysis. A statistically significant difference was also observed in the Kaplan–Meier analysis (log-rank test, *p* = 0.021). However, sex was not an independent prognostic factor for recurrence in multivariate analysis. Several studies have reported that male sex was not an independent risk factor for survival in DTC^20,37^. After matching, our analyses confirmed that sex was not a significant predictor of DTC recurrence.

It is difficult to explain why the DFS did not significantly differ between male and female patients with DTC in current study. However, this result may be attributed to the long-term decreasing prognostic potential of male sex. Choi et al. have assessed secular trends in the prognostic factors for PTC. They reported that the risk for poor outcomes associated with male sex decreased over time in PTC. However, the risk associated with pathologic characteristics remained the same or increased over time^21^. Further, another study has shown that young women had a better prognosis, and the outcomes were similar between men and those aged > 55 years^33^. Therefore, a subgroup analysis was conducted to evaluate the differences in prognosis between male and female patients according to age. The patients were divided into two groups, which were as follows: young age group (≤ 45 years) and old age group (> 45 years). We validated that sex was not an independent prognostic factor for recurrence in both age groups in the multivariate analysis after propensity score matching. The cause of the difference in DTC presentation between males and females is unclear. However, the increased number of screening tests might be correlated to these changes. In the past, men were less likely to visit hospitals than women; thus, they present with more aggressive stages of thyroid cancer. In addition, since a high proportion of women present with benign thyroid disease, such as thyroiditis, screening is faster due to serial follow-up. Therefore, women with thyroid cancer could be diagnosed at earlier stage. However, with the higher number of health examinations performed, the number of male patients with incidentally detected thyroid cancer has been increasing recently. Machens et al. emphasized the need for earlier diagnosis and intervention in men to validate whether male sex is an ominous prognostic factor of advanced-stage thyroid cancer^12^.

Another study showed that sex was an age-specific effect modifier for the incidence of PTC. Thus, the sex gap in the incidence of PTC decreased with age^33,38^. Since the proportion of elderly male patients is relatively higher, age might be an important confounding factor when conducting a comparison between two groups according to sex. Hence, sex might not be an independent prognosis factor^39^.

Based on the results of our research, large multicenter studies with propensity score matching on sex difference should be conducted to determine if our results are confirmed. That is, sex is not an independent prognosis factor. To date, most studies, such as that conducted by Zahedi et al., did not perform propensity score matching^18^. In addition, we might conduct a study with a subgroup analysis according to tumor size. Lee et al. showed that male sex is an independent prognostic factor for recurrence in PTC > 1 cm, but not in thyroid microcarcinoma^19^. By conducting an analysis using propensity score matching on whether sex has a tumor size-specific effect, further research can identify whether the management of males and females should vary based on tumor size.

This study had several limitations. That is, it was retrospective in nature. Moreover, there might have been selection bias because data were collected at a single tertiary institution and do not represent the entire patient population. However, propensity score matching was performed to adjust for differences in clinicopathological characteristics and to minimize bias. In propensity score matching analysis, data loss from 3486 patients should also be considered. Finally, the mean follow-up period was relatively short (99.9 ± 18.7 months). Hence, a longer follow-up is required to predict the prognosis of patients with DTC, as it has indolent characteristics. In addition, although all patients were followed up for the appropriate levothyroxine dose, RAI ablation, and serum Tg level, those parameters were not appropriately included in this study. Because these parameters are crucial and may affect the propensity score matching, more reliable results would have been obtained if the above parameters were included.

This study had several important strengths. Each patient was followed up, and a standardized laboratory and imaging protocol was used in a single institution. Moreover, this is one of the largest cohort studies conducted on this topic to date. To the best of our knowledge, few studies have used propensity score matching to evaluate the effect of sex on the long-term prognosis for DTC.

In conclusion, compared with females, male patients were more likely to present with more aggressive disease characteristics, including larger tumor size, higher prevalence of ETE and lymphatic and vascular invasions, greater proportion of positive LNs, higher grade of TNM stage, and higher recurrence rate. However, after propensity score matching, no significant difference was observed in the recurrence rates between the matched groups. Therefore, male sex was not found to be an independent prognostic factor for DTC recurrence. Further studies with larger cohorts should be conducted to validate this result.

## Methods

### Patients

We retrospectively analyzed 5827 patients with DTC who underwent thyroid surgery from January 2009 to December 2015 at Seoul St. Mary’s Hospital (Seoul, Korea). In total, 198 patients with incomplete data and 63 who were lost to follow-up were excluded from the analysis. The medical charts and pathology reports of 5566 patients were reviewed and analyzed. According to sex, patients were categorized into two groups (female and male groups). The mean follow-up duration was 99.9 ± 18.7 (range 66–137) months. This study was conducted in accordance with the Declaration of Helsinki (as revised in 2013) and was approved by the institutional review board of Seoul St. Mary’s Hospital, The Catholic University of Korea (IRB No: KC20RISI0829), The need for informed consent was waived due to the retrospective nature of this study.

### Preoperative workup

The preoperative workup included physical examination, serum thyroid function test, neck ultrasonography (US), and computed tomography (CT) scan. Thyroid nodules were diagnosed based on preoperative US-guided fine needle aspiration cytology (FNAC). FNAC was performed by experienced radiologists. The diagnoses made by using FNAC were based on the Bethesda system^40^. To validate the size and location of tumors, presence/absence of ETE, LN status, and other abnormal findings in the neck, all patients diagnosed with DTC were evaluated via preoperative US and neck CT scan. If there were palpable LNs or suspicious LNs were found on preoperative US or neck CT, FNAC and washout thyroglobulin testing were performed. The surgical extent was decided in accordance with the ATA management guideline.

### Postoperative management and follow-up

Postoperative care and follow-up were conducted according to the ATA management guidelines regardless of sex. Patients underwent physical examination, neck US, and serum thyroid function testing at 3–6-month intervals and annually thereafter. All patients took suppressive doses of L-thyroxine and were regularly followed up by physical examination, thyroid function testing, Tg and anti-Tg antibody concentration measurements, and neck US at 3–6-month intervals and annually thereafter. Radioactive iodine (RAI) ablation was performed at 6–8 weeks postoperatively, and whole body scans were performed at 5–7 days after RAI ablation in patients who underwent total thyroidectomy.

Those with recurrence or distant metastasis on routine follow-up evaluations underwent additional diagnostic imaging, including CT scan, positron emission tomography/CT scan, and/or radioactive iodine whole body scan, to determine the location and extent of suspected recurrence. In cases of suspected recurrence in the remnant thyroid, resection bed, or lymph nodes, diagnosis was confirmed via histologic examination using FNAC or a surgical biopsy specimen.

### Primary and secondary endpoints

The primary endpoint was DFS between males and females after propensity score matching, and the secondary endpoint was the clinicopathological characteristic differences between males and females before and after propensity score matching.

### Statistical analysis

Continuous variables were presented as means with standard deviations and categorical variables were presented as numbers with percentages. Student’s *t*-test and the Pearson’s chi-square test or Fisher’s exact test were used to compare continuous and categorical variables, respectively. Univariate and multivariate Cox regression analyses were performed to validate the predictors of DFS, and HRs with 95% CIs were calculated. DFS was compared by performing Kaplan–Meier survival analysis with the log-rank test.

To reduce the effects of selection bias and potential confounding factors, propensity score matching was performed using various clinicopathological characteristics: age, extent of operation, tumor size, ETE, lymphatic invasion, vascular invasion, harvested LNs, positive LNs, N stage, and TNM stage. Individual patient propensity scores were calculated via logistic regression analysis. After propensity score matching, the clinicopathological characteristics representative of long-term oncologic outcome and recurrence were compared between females and males. A *p* value of < 0.05 was considered statistically significant. All statistical analyses were performed by using the Statistical Package for the Social Sciences software for Windows version 23.0 (IBM SPSS Statistics for Windows, IBM Corp., Armonk, NY).

## Data Availability

The data that support the findings of this study are available on request from the corresponding author. The data are not publicly available due to privacy or ethical restrictions.
